# Psychological Stress Modulates Bone Remodeling Pathways in Normotensive and Hypertensive Rats: A Cellular and Molecular Approach

**DOI:** 10.1021/acsomega.5c11197

**Published:** 2026-02-19

**Authors:** Marina Ribeiro Paulini, Dimitrius Leonardo Pitol, Glauce Crivelaro do Nascimento, Daniela Vieira Buchaim, Marcelo Rodrigues da Cunha, Rogerio Leone Buchaim, João Paulo Mardegan Issa

**Affiliations:** † Department of Basic and Oral Biology, School of Dentistry of Ribeirão Preto, University of São Paulo (FORP-USP), Ribeirão Preto 14040-904, Brazil; ‡ Medical School, University Center of Adamantina (FAI), Adamantina 17800-000, Brazil; § Graduate Program in Anatomy of Domestic and Wild Animals, Faculty of Veterinary Medicine and Animal Science, University of São Paulo (FMVZ-USP), São Paulo 05508-270, Brazil; ∥ Department of Postgraduate, Dentistry School, Faculty of the Midwest Paulista (FACOP), Piratininga 17499-010, Brazil; ⊥ Postgraduate Program in Health Sciences, Faculty of Medicine of Jundiaí (FMJ), Jundiaí 13202-550, Brazil; # Department of Biological Sciences, Bauru School of Dentistry (FOB/USP), University of Sao Paulo, Bauru 17012-901, Brazil

## Abstract

Hypertension and psychological stress are prevalent conditions that may adversely affect bone health, yet their combined impact on bone remodeling remains underexplored. This study aimed to investigate the effects of acute and chronic stress on bone structure and remodeling in normotensive and spontaneously hypertensive rats. Forty male rats (20 normotensive Hannover and 20 spontaneously hypertensive) were randomly assigned to control, acute stress, or chronic variable stress groups. Acute stress consisted of a single 2 h restraint session, while chronic variable stress involved five different stressors over 10 days. Bone remodeling was assessed using quantitative histology, immunohistochemistry for VEGF, bone sialoprotein (BSP), osteocalcin (OCN), and TRAP, as well as enzymatic activity analysis (MMP-2 and MMP-9). Results: Neither acute nor chronic stress significantly altered trabecular bone volume or collagen density within the study period. However, stress exposure reduced osteoblast activity (OCN, BSP), increased matrix remodeling enzymes (MMP-2, MMP-9), and promoted angiogenic signaling (VEGF). Notably, TRAP-positive osteoclasts were observed exclusively in stress-exposed animals, indicating elevated bone resorption. Importantly, hypertensive rats exhibited inherently higher basal levels of remodeling activity and MMP-2 expression compared to normotensive controls, and showed a more pronounced response to chronic stress, suggesting hypertension may predispose bone tissue to increased metabolic vulnerability. According to the results of corticosterone dosage, as well as histological and immunohistochemical analyses, it is provided that stress promotes changes in bone metabolism, corroborating the hypothesis that its effects occur early and precede detectable structural modifications. These findings indicate that both hypertension and psychological stress are relevant modulators of bone physiology, and that their interaction may amplify metabolic changes in bone remodeling, emphasizing the importance of monitoring bone health in hypertensive individuals exposed to stress.

## Graphical abstract

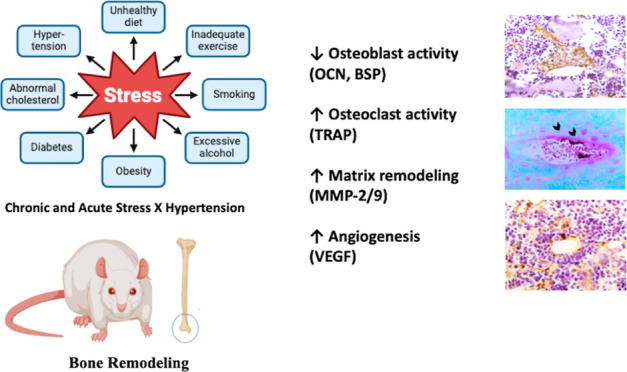

## 1 Introduction

Hypertension is among the most prevalent cardiovascular disorders and stands as a leading cause of mortality worldwide, commonly associated with adverse outcomes such as stroke and acute myocardial infarction.^1^ Multiple factors contribute to its development, including obesity, smoking, alcohol consumption, family history, personality traits, and psychological elements such as stress. Both genetic predisposition and environmental influences—such as physical inactivity and high sodium intake—are also well-established contributors.^2–5^

Although hypertension has been widely studied, the influence of emotional and psychological factors—such as stress, anxiety, hostility, and impulsivity—on blood pressure regulation has received comparatively less attention.^6–8^ It is proposed that stress triggers sympathetic nervous system activation, which in turn elevates blood pressure levels.^9–11^ Stressful life events, including interpersonal disputes, financial difficulties, and social isolation, can elicit physiological and behavioral changes that disrupt neuroendocrine and immune system function, contributing to diverse individual responses.^12–18^ The skeletal system is highly dynamic, undergoing continuous remodeling to ensure structural integrity and mineral balance. Mechanical stimuli are essential for promoting bone formation and preventing bone loss through osteogenic signaling pathways.^19,20^

Interestingly, hypertension has been associated with disturbances in calcium metabolism and increased bone resorption, factors that may contribute to decreased bone mineral density in affected individuals.^21–23^ As global populations age and lifestyle-related factors—such as sedentary behavior, psychological stress, and poor dietary habits—become more prevalent, the incidence of hypertension and osteoporosis has markedly increased.^24–26^ These conditions often coexist and are shaped by intricate interactions between genetic predispositions and environmental influences.^27,28^

In a previous pilot study, we performed qualitative histological analysis and microcomputed tomography (micro-CT)^29^ of long bones in spontaneously hypertensive rats, providing preliminary insights into the morphofunctional characteristics of bone under hypertensive conditions. Stress is thought to influence bone homeostasis through neuroendocrine mechanisms, including glucocorticoid-mediated suppression of osteoblast activity and sympathetic signaling that increases osteoclastogenesis, which may exacerbate hypertensive bone alterations. Building on these findings, the present study was designed to complement and extend this work by examining the influence of acute and chronic stress on bone remodeling, with additional assessment of cellular and matrix-related markers through quantitative histology and immunohistochemistry, including VEGF, bone sialoprotein, osteocalcin and TRAP.

In light of the growing burden of chronic diseases and emotional stress across populations, there is an increasing demand for integrative research addressing the physiological, morphological, and biochemical alterations associated with these health challenges.^30,31^ Therefore, the aim of this study is to investigate the effects of acute and chronic stress, in combination with hypertension, on the skeletal system. In line with this objective, the null hypothesis tested is that acute and chronic stress, alone or combined with hypertension, do not promote significant changes in bone remodeling markers. Quantitative histological analysis and immunohistochemistry will be performed, including the evaluation of VEGF, bone sialoprotein, osteocalcin, and TRAP markers.

## 2 Materials and Methods

### 2.1 Ethical Approval and Experimental Design

This study was approved by the Animal Research Ethics Committee of the University of São Paulo, School of Dentistry of Ribeirão Preto, Brazil (Protocol No. 0027/2021R2). A total of 40 male rats were used (6 month-old), comprising 20 normotensive Hannover rats and 20 spontaneously hypertensive rats (SHR), each weighing approximately 250 g at baseline. All animals were obtained from the Central Animal Facility of the University of São Paulo, Ribeirão Preto campus. The animals were housed individually in standard cages, under controlled environmental conditions (24 ± 1 °C, 12 h light/dark cycle, lights on at 07:00 AM), with ad libitum access to food and water. All experimental procedures were conducted during the morning hours in a quiet, temperature-controlled room to minimize variability. Daily monitoring included manual recording of body weight, food and water intake, and cage maintenance, performed consistently at 08:00 AM during the light cycle.

The animals remained unaffected during the dark phase. The rats were randomly assigned to the experimental groups: G1 - Control (normal rats, control); G2 - Control (SHRs, control); G3 - Acute stress/control (acute stress control/normal rats); G4 - Acute stress (normal rats subjected to acute stress); G5 - Acute stress/control (acute stress control/SHRs); G6 - Acute stress (SHRs subjected to acute stress); G7 - Chronic stress/control (chronic stress control/normal rats); G8 - Chronic stress (normal rats subjected to chronic stress); G9 - Chronic stress/control (chronic stress control/SHRs); and, G10 - Chronic stress (SHRs subjected to chronic stress). Group G3 corresponds to the control group of normal animals subjected to acute stress. That is, these animals did not experience acute stress but remained in cages until all other animals in the acute stress groups were euthanized. The same occurred in group G5, but for SHR animals. Group G7 corresponds to the control group of normal animals subjected to chronic stress. These animals did not undergo the 10 day chronic stress period but remained in cages until all other animals in the chronic stress groups were euthanized. The acute and chronic stress groups were divided into control groups and exposed to stress to ensure that the observed effects could be attributed specifically to the stress protocol and not to other experimental variables, such as handling, time in the facility, or environmental exposure. The internal control groups (G3, G5, G7, G9) provided a direct reference for the stressed animals within each group, complementing the control groups G1 and G2, which represent only the baseline condition of normotensive and hypertensive rats without any stress intervention. The minimum number of animals per group was determined through an a priori power analysis performed using G*Power software (version 3.1.9.7, Universität Düsseldorf, Germany). Based on pilot data and previous studies conducted by our research group using similar stress models and hypertensive rats,^32–34^ we assumed a large expected effect size (*f* = 0.50), estimated variance of approximately 10%, type I error α = 0.05, and statistical power (1−β) = 0.80. Considering one-way ANOVA with ten experimental groups, the calculation indicated a minimum requirement of *n* = 4 rats per group, resulting in a total sample size of *N* = 40 animals. A conservative number of subjects was adopted to prevent potential losses during the stress protocols; however, no animals died or were excluded throughout the experimental period (Table 1) (Annex A).

**Table 1:** Distribution of Hannover Normotensive and Spontaneously Hypertensive Rats (SHR) Across the Experimental groups and stress Conditions^a^

group ID	condition	strain	stress exposure	description	*n*
G1	control baseline	normotensive (hannover)	none	normal control	4
G2	control baseline	spontaneously hypertensive rats (SHR)	none	hypertensive control	4
G3	acute control	normotensive	no stress (remained in cage)	internal control for acute protocol	4
G4	acute stress	normotensive	2 h physical restraint	acute stress – normal	4
G5	acute control	SHR	no stress (remained in cage)	internal control for acute protocol	4
G6	acute stress	SHR	2 h physical restraint	acute stress – SHR	4
G7	chronic control	normotensive	internal control – remained in cage	matched for chronic stress	4
G8	chronic stress	normotensive	10 day CVS	chronic stress – normal	4
G9	chronic control	SHR	internal control – remained in cage	matched for chronic stress	4
G10	chronic stress	SHR	10 day CVS	chronic stress – SHR	4
total					40 rats

a Each group comprised four animals (*n* = 4), totaling 40 rats. Internal control groups (G3, G5, G7, and G9) were housed under the same environmental and handling conditions as stressed rats but were not exposed to stress, serving as reference controls for acute and chronic comparisons.

### 2.2 Acute Stress Protocol

A total of 16 rats (both normotensive and hypertensive) were subjected to an acute stress paradigm involving a single session of physical restraint lasting 2 h. Each animal was placed in a ventilated cylindrical metal restrainer (15 cm in length × 5 cm in diameter), where movement was restricted. The stress session was performed once in the morning under standardized conditions.^32–34^

### 2.3 Chronic Variable Stress Protocol

Chronic variable stress (CVS) was used to simulate unpredictable, real-life stressors experienced daily by individuals. The protocol was adapted from previously published work by our research group.^32–34^ Five different stressors were applied over a 10 day period, with each stressor repeated on two nonconsecutive days.

All procedures were performed in the morning, as follows: days 01 and 06 – agitation stress: rats were placed individually in plastic containers on a shaker platform set at 50 rpm for 15 min. Days 02 and 07 – forced swimming: animals swam individually for 15 min in a circular plastic tank (54 cm height × 47 cm diameter) filled with water to a depth of 40 cm, maintained at 25 ± 1 °C, preventing contact with tank walls or bottom. Days 03 and 08 – physical restraint: As in the acute protocol, rats were confined in metal restrainers (15 cm × 5 cm) for 2 h. Days 04 and 09 – cold exposure: rats were exposed to cold-induced stress by placing them in individual plastic cages in a refrigeration unit (10 °C) for 30 min. Days 05 and 10 – water deprivation: animals were deprived of water for 24 h. A schematic representation of the chronic stress protocol is shown in Figure 1.

**Figure 1:**
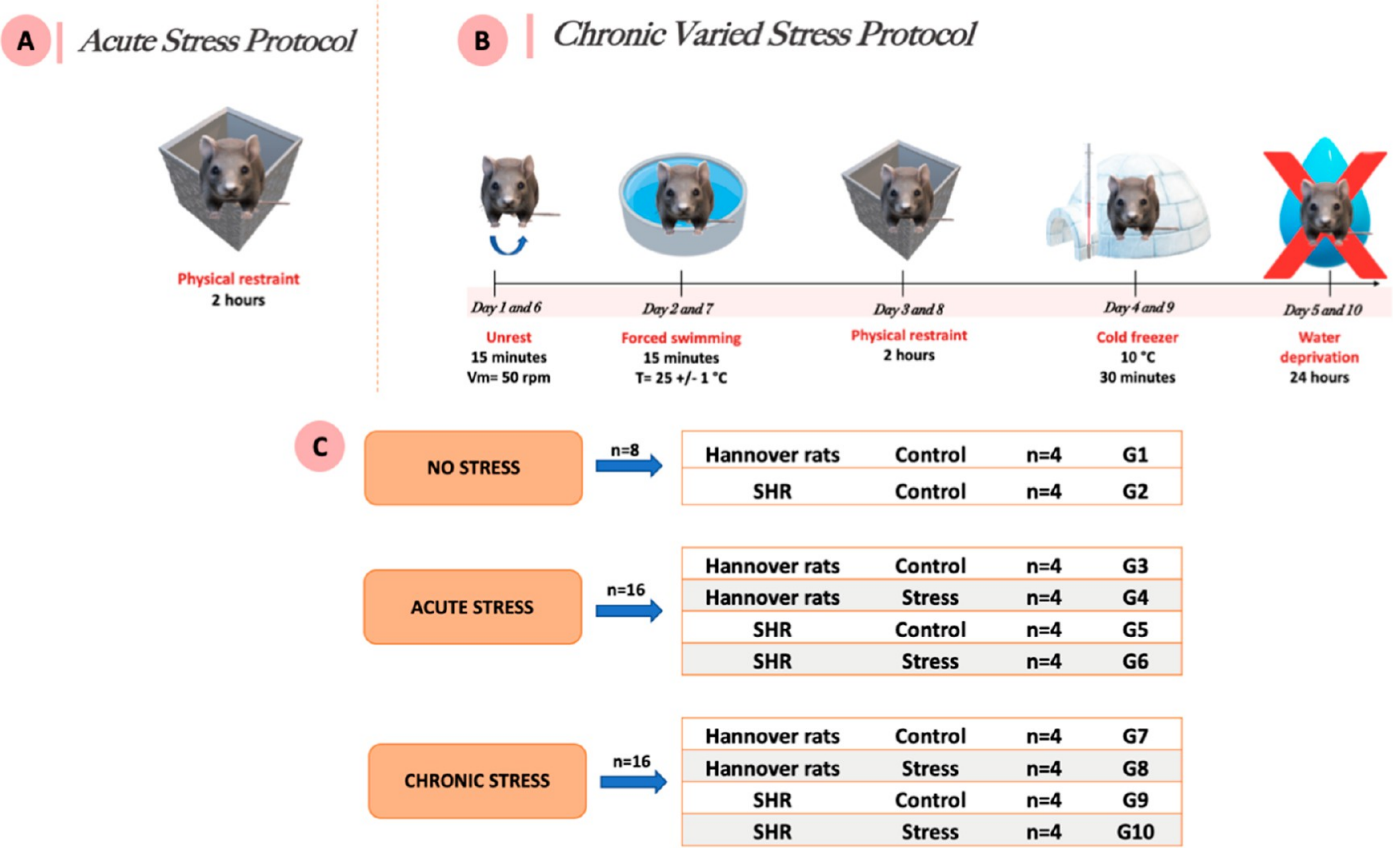
Experimental design of stress protocols - (A) acute stress protocol: single episode of physical restraint stress for 2 h. (B) Varied chronic stress protocol. (C) Division of experimental groups: G1-control (normal rats, control); G2-control (SHRs, control); G3-control (normal rats, acute stress control); G4-acute stress (normal rats subjected to acute stress); G5-control (SHRs, acute stress control); G6-acute stress (SHRs subjected to acute stress); G7-control (normal rats, chronic stress control); G8-chronic stress (normal rats subjected to chronic stress); G9-control (SHRs, chronic stress control); and, G10-chronic stress (SHRs subjected to chronic stress). Figure created by the authors using BioRender.com.

### 2.4 Tibia Collection

At the end of the experiment, the animals were euthanized, having been previously anesthetized with 4% xylazine (14 mg/kg) and 10% ketamine (100 mg/kg), via intraperitoneal injection, and were subjected to euthanasia by decapitation 24 h after the last stress exposure. In rats subjected to acute stress, euthanasia was performed immediately after the stress. Subsequently, the right and left tibias of each animal were removed, dissected, and fixed in 10% formaldehyde phosphate buffer (pH 7.4) for 48 h.

### 2.5 Corticosterone Hormone

Corticosterone levels were measured from plasma obtained at the time of animal euthanasia by decapitation. All euthanasia and blood collections were performed in the morning (between 08:00 and 10:00 a.m.) to control for circadian fluctuations in corticosterone levels. Blood was collected into heparinized tubes and centrifuged in a refrigerated centrifuge at 4 °C, at 3500 rpm for 20 min. The resulting plasma supernatant was transferred to cryotubes and stored at −80 °C until analysis. Corticosterone concentrations were determined using an enzyme-linked immunosorbent assay (ELISA), following the manufacturer’s instructions.

### 2.6 Histological Processing and Quantitative Analysis

Right tibias were harvested, fixed in 10% buffered formaldehyde for 24–48 h, and decalcified in 10% EDTA under constant agitation for approximately 15 days, until complete decalcification was achieved. Specimens were embedded in paraffin using Histosec polymer (Merck KGaA, Darmstadt, Germany), and 5 μm longitudinal sections were obtained and stained with hematoxylin and eosin (H&E) for general morphological evaluation, Masson’s trichrome for bone matrix organization and Picro Sirius Red for collagen fiber evaluation. For Picro Sirius Red staining, sections were deparaffinized, hydrated, and incubated with Picro Sirius Red solution (0.1% Sirius Red F3B in saturated aqueous picric acid) for 60 min, followed by washing in acidified water, dehydration, and mounting. Collagen fibers were visualized under polarized light microscopy, and images were captured as described below.

Slides were coded according to experimental groups, and all evaluations were performed by a blinded examiner to minimize observer bias. Images were captured using a digital camera attached to a Zeiss AxioImager Z2 microscope (Zeiss, Oberkochen, Germany) with standardized settings, at ×10, ×20, and ×40 magnifications. For each animal, five slides were analyzed, totaling 15 images per animal. Across 10 experimental groups with four animals each, 600 images were analyzed. Quantitative analysis was performed using AxioVision 4.8 software (Carl Zeiss, Oberkochen, Germany) following technical calibration. The software identified blue-stained bone areas (Masson) and collagen fibers (Picro Sirius Red), and results were expressed as the percentage of area relative to total area, focusing on the distal region of the tibia. These measurements enabled comparisons across experimental groups (Figure 2).

**Figure 2:**
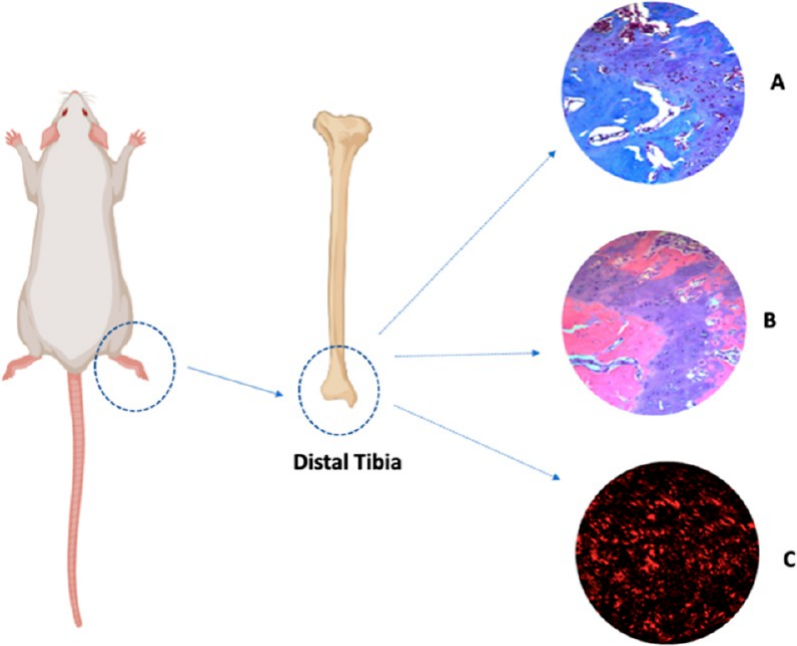
Schematic illustration of the experimental animal highlighting the distal tibia region, used for the definition of the region of interest. (A) Image stained with Masson’s trichrome. (B) Image stained with hematoxylin and eosin. (C) Image stained with Picro Sirius red. Figure created by the authors using BioRender.com.

### 2.7 Immunohistochemical Analysis

After histological sectioning, the previously silanized odd-numbered slides were selected for immunohistochemical analysis. Five slides were used per animal. The proteins analyzed included vascular endothelial growth factor (VEGF), bone sialoprotein (BSP), and osteocalcin (OCN). The following primary antibodies were used: Rabbit anti-VEGF monoclonal antibody (Abcam, code ab12345, dilution 1:100), rabbit anti-BSP polyclonal antibody (Abcam, code ab67890, dilution 1:100), and mouse antiosteocalcin monoclonal antibody (Abcam, code ab13579, dilution 1:200), according to the manufacturer’s protocol. The procedure followed standardized steps: deparaffinization and rehydration of the slides, followed by endogenous peroxidase blocking with hydrogen peroxide for 10 min.

After buffer washing, antigen retrieval was performed when necessary, followed by blocking of nonspecific binding with protein block and incubation with the primary antibody, according to the manufacturer’s instructions. Subsequently, a secondary reagent (complement) specific for the primary antibodies was applied, followed by incubation with goat antirabbit HRP-conjugated antibody for 15 min. Detection was carried out using DAB chromogen, with an incubation time ranging from 1 to 10 min. The slides were counterstained, dehydrated, and mounted with coverslips. For the histochemical analysis of tartrate-resistant acid phosphatase (TRAP) activity, bone fragments were initially decalcified in 10% EDTA solution, followed by fixation in 10% buffered formalin for 24 to 48 h.

After further decalcification in EDTA under constant agitation, the tissues were embedded in paraffin and semiserially sectioned in a microtome at 5 μm thickness, yielding two nonconsecutive longitudinal sections per animal (with a minimum distance of 100 μm between sections). Immunohistochemical analysis was performed using five slides per marker (VEGF, osteocalcin, bone sialoprotein, and TRAP) resulting in a total of 20 images per experimental group, five images per immunohistochemical reaction. The slides were deparaffinized in xylene (10 min per bath) and absolute ethanol (three 5 min baths), followed by incubation for 40 min at 37 °C in a solution of naphthol AS-MX phosphate, sodium acetate anhydrous, and l-(+)-tartaric acid (Sigma).

The reaction was stopped in distilled water, and counterstaining was performed with 1% Fast Green for 1 min, followed by dehydration, clearing in xylene, and mounting in Entellan. TRAP enzymatic activity was histochemically analyzed in the distal region of the tibia. Images were acquired using an AxioImager Z2 light microscope (Carl Zeiss, Oberkochen, Germany), equipped with a digital camera and AxioVision 4.8 software. TRAP-positive osteoclasts were defined as multinucleated cells (two or more nuclei) in direct contact with the bone surface, observed under a 40× objective and a 50 μm field.^34^

### 2.8 Zymography for MMP-2 and MMP-9 Activity

Gelatin zymography was performed on left tibias to evaluate the enzymatic activity of matrix metalloproteinases MMP-2 and MMP-9 in bone tissue from all experimental groups. After euthanasia, tibiae were collected, cleaned of soft tissue, and immediately frozen at −80 °C. Bone samples were then pulverized under liquid nitrogen and homogenized in ice-cold lysis buffer containing 50 mM Tris-HCl (pH 7.6), 150 mM NaCl, 1% Triton X-100, 0.1% SDS, and protease inhibitors. Protein concentration was determined using the Bradford assay. Equal amounts of protein (30 μg per lane) were mixed with nonreducing sample buffer and loaded onto 10% SDS-PAGE gels copolymerized with 0.1% gelatin.

Electrophoresis was performed at 4 °C. Gels were then washed twice in 2.5% Triton X-100 for 30 min to remove SDS and incubated overnight at 37 °C in activation buffer containing 50 mM Tris-HCl (pH 7.6), 5 mM CaCl_2_, and 0.02% NaN_3_. Following incubation, gels were stained with 0.5% Coomassie Brilliant Blue R-250 for 1 h and subsequently destained in a solution of 10% acetic acid and 30% methanol until clear bands corresponding to gelatinolytic activity were visible against the blue background. Bands representing pro-, intermediate, and active forms of MMP-2 and intermediary MMP-9 were quantified by densitometry using ImageJ software, and results were expressed in arbitrary units of optical density.

This methodology allowed for the detection and comparison of MMP enzymatic activity among the different experimental groups, providing insights into the remodeling dynamics of the bone extracellular matrix under acute and chronic stress conditions.

### 2.9 Statistical Analysis

The percentage of newly formed bone assessed by quantitative histology, as well as serum corticosterone levels, bone resorption, and collagen fiber density, were subjected to statistical analysis. Data normality was evaluated using the Kolmogorov–Smirnov test, and homogeneity of variances was assessed using Bartlett’s test. One-way ANOVA was then applied, followed by Tukey’s post hoc test for group comparisons. Statistical analyses were performed using GraphPad Prism software (version 8.0, GraphPad Software, La Jolla, CA, USA), with a significance level set at 5% (*p* < 0.05).

## 3 Results

### 3.1 Plasmatic Corticosterone Dosage

Serum corticosterone levels were measured in all experimental groups (G1 to G10). The means and standard deviations are presented in Table 2 and illustrated in Figure 3. Statistical analysis indicated significant differences among the groups (*p* < 0.05). Groups G3 to G6 showed significantly higher corticosterone levels compared to group G1 (*), indicating a robust physiological response to acute or chronic stress. Among these, group G6 exhibited the highest mean concentration (71.3 ± 15.3 ng/mL), suggesting intensified activation of the hypothalamic-pituitary-adrenal (HPA) axis. On the other hand, groups G7 to G10 also showed significantly increased levels compared to group G2 (#), although with values lower than those observed in G6.

**Table 2:** Serum corticosterone Levels in Experimental groups (Mean ± Standard Deviation, ng/mL)

group	corticosterone (ng/mL)
G1	16.5 ± 1.7
G2	26.0 ± 3.2
G3	57.3 ± 5.9
G4	54.3 ± 7.8
G5	58.8 ± 7.8
G6	71.3 ± 15.3
G7	48.3 ± 3.0
G8	47.0 ± 6.5
G9	54.8 ± 3.0
G10	49.0 ± 9.8

**Figure 3:**
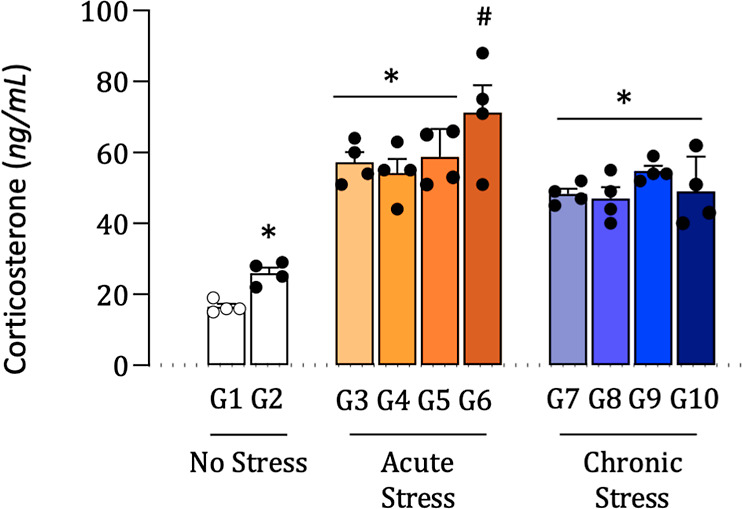
Serum corticosterone levels (ng/mL) in experimental groups (G1–G10). Data are presented as mean ± standard deviation. * Indicates statistically significant difference compared to group G1 (normotensive control); # indicates significant difference compared to group G2 (hypertensive control); One-way ANOVA followed by Tukey’s post hoc test, *p* < 0.05.

### 3.2 Quantitative Histology Results and Total Collagen Fibers

Quantitative histomorphometric analysis of bone tissue (BA/TA, %) revealed mean values ranging from 8.54% (G4) to 10.85% (G5) (Figure 4A). The normal control group (G1) presented a mean of 10.52%, similar to G2 (10.47%) and to most of the other groups, with no consistent trend toward an increase or decrease in bone area. However, a slight difference was observed between groups G2 and G9.

**Figure 4:**
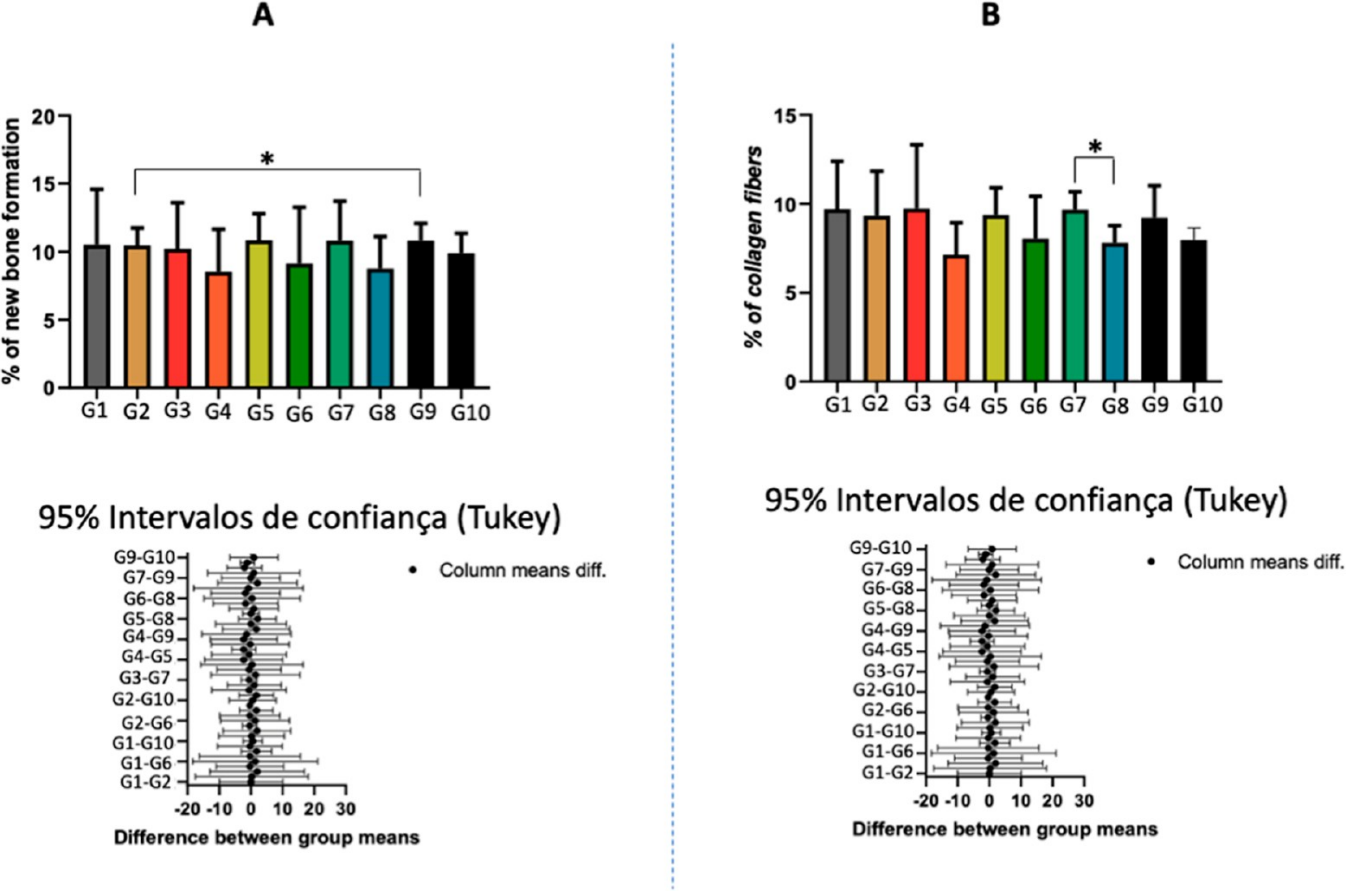
Quantitative histological analysis of bone tissue and total collagen fibers. (A) Percentage of bone area (BA/TA, %) in the experimental groups. A slight difference was observed between G2 (SHR control) and G9 (chronic stress/normal rat). (B) Percentage of total collagen fibers assessed by Picro Sirius Red staining under polarized light microscopy. A discrete difference was observed between G7 (chronic stress/SHR) and G8 (chronic stress/normal rat). Data are presented as mean ± SD. Statistical analysis was performed using repeated-measures ANOVA followed by Tukey’s post hoc test (*p* < 0.05).

Total collagen fiber quantification, assessed by Picro Sirius Red staining under polarized light, showed mean values between 7.15% (G4) and 9.72% (G3) (Figure 4B). The normal control group (G1) recorded 9.71%, and the SHR control group (G2) 9.33%, with comparable values across groups overall. Nonetheless, although group G7 presented higher values than group G8, no statistically significant difference was observed.

For both variables (BA/TA % and collagen density), five nonoverlapping microscopic fields per histological section were analyzed for each animal, representing repeated measurements within the same specimen. Accordingly, repeated-measures ANOVA was applied, considering the animal as the experimental unit. The analysis revealed no statistically significant differences between groups for bone area (*F*(1.672, 6.687) = 0.4081; *p* = 0.6463) or total collagen fibers (*F*(2.063, 8.254) = 1.028; *p* = 0.4017). Altogether, these findings indicate that, despite minor variations between groups, the experimental protocols did not significantly influence bone formation or collagen deposition.

In addition to the quantitative data, qualitative histological analysis provided further insights into bone tissue characteristics (Figure 5). Masson’s Trichrome and H&E staining revealed the distribution of bone marrow (yellow arrows) and growth plate/cartilage (red arrows), while Picro Sirius Red staining under polarized light microscopy highlighted total collagen fibers (white arrows). These qualitative observations complement the morphometric data, illustrating the preservation of trabecular structures, cartilage regions, and collagen distribution across the experimental groups.

**Figure 5:**
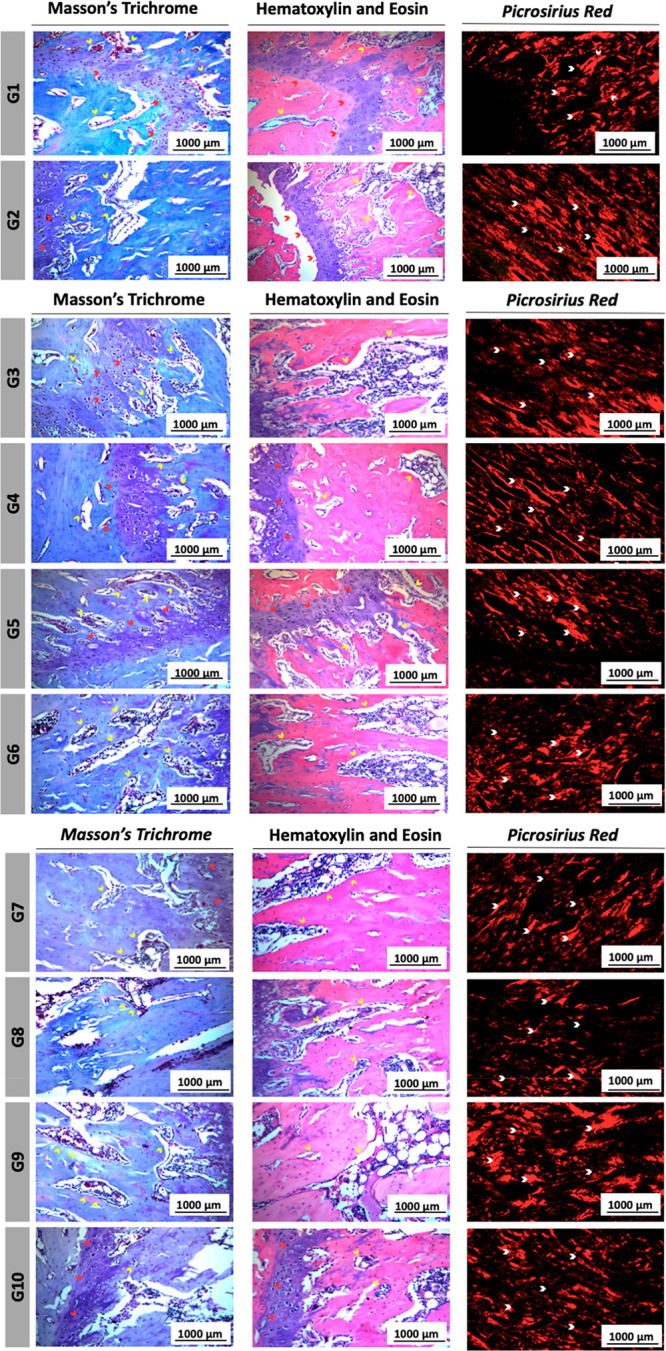
Histological analysis of bone tissue from experimental groups (G1–G10). Representative images stained with Masson’s trichrome, hematoxylin and eosin (H&E), and Picro Sirius Red. In Masson’s trichrome and H&E, yellow arrows indicate bone marrow and red arrows indicate the cartilaginous growth plate. In Picro Sirius Red staining, white arrows indicate total collagen fibers, visualized under polarized light microscopy. Scale bar: 1000 μm.

### 3.3 Qualitative Results of Immunohistochemistry (Osteocalcin, Bone Sialoprotein, VEGF, TRAP)

Immunohistochemical analysis was performed to evaluate the expression of osteocalcin (OCN), bone sialoprotein (BSP), and vascular endothelial growth factor (VEGF) in bone tissue from the different experimental groups (G1 to G10).

Osteocalcin (OCN) is a noncollagenous protein primarily synthesized by osteoblasts and is directly involved in the bone mineralization process, serving as an important marker of bone-forming activity. In this study, strong osteocalcin immunostaining was observed in areas of osteoblastic activity in the control groups (G1 and G2), with widespread distribution in both osteoblasts and the mineralized bone matrix. In contrast, the groups subjected to acute and chronic stress protocols (G3 to G10) showed a visible reduction in staining intensity and in the number of OCN-positive cells, indicating decreased osteoblastic activity in these groups (Figure 6).

**Figure 6:**
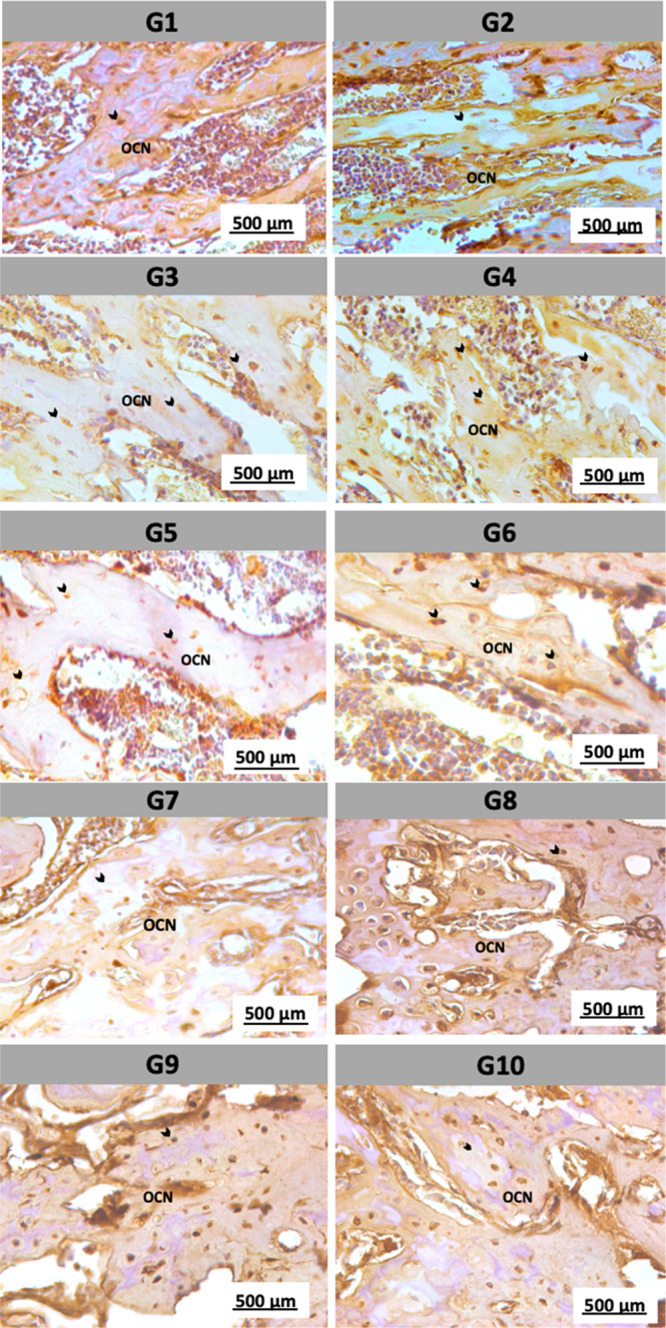
Immunohistochemical expression of osteocalcin (OCN). Intense immunostaining for osteocalcin was observed in the control groups (G1 and G2), with widespread distribution in osteoblasts and mineralized bone matrix, reflecting high osteoblastic activity. The black arrows indicate osteocytes embedded in the bone matrix and in certain bone marrow cells. The groups subjected to the acute and chronic stress protocols (G3 to G10) showed a reduction in staining intensity and in the number of positively stained cells, suggesting decreased bone formation activity. Scale bar: 500 μm.

Bone sialoprotein (BSP), another noncollagenous component of the bone matrix, is essential for hydroxyapatite nucleation and cell adhesion, playing a key role in bone matrix mineralization and integrity. In our study, BSP showed strong and diffuse expression in the control groups, particularly in bone mineralization regions, with intense and widespread staining. In the stress-exposed groups, BSP expression was clearly reduced, with decreased staining intensity and distribution, suggesting altered matrix quality and potential impairment of the mineralization process (Figure 7).

**Figure 7:**
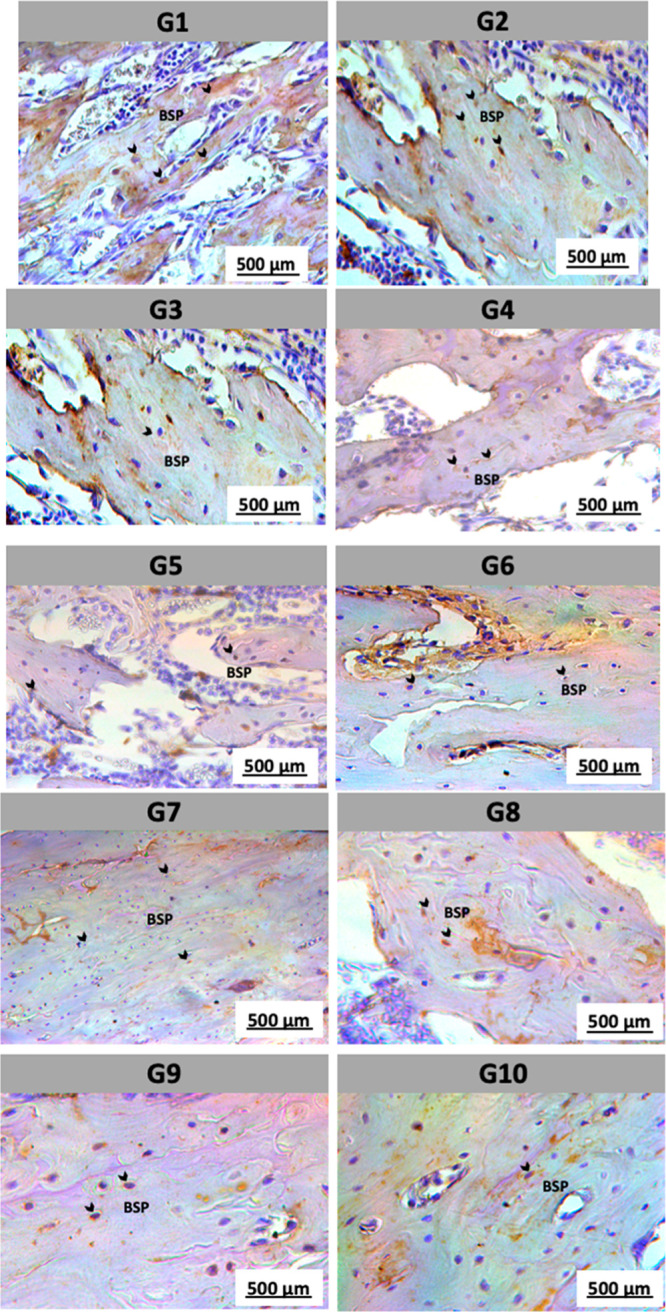
Immunohistochemical expression of bone sialoprotein (BSP). Strong and diffuse immunostaining for BSP was observed in the control groups (G1 and G2), mainly in the mineralized bone regions, reflecting preserved mineralization activity and matrix integrity. In contrast, the groups exposed to acute and chronic stress protocols (G3 to G10) showed a marked reduction in staining intensity and area, suggesting alterations in bone matrix quality and potential impairment of mineralization processes. Black arrows indicate areas of positive BSP staining. Scale bar: 500 μm.

Lastly, VEGF is a growth factor that regulates angiogenesis, promoting the formation of new blood vessels, which is crucial for nutrient supply and bone remodeling. VEGF expression varied among the experimental groups. In the control groups (G1 and G2), VEGF staining was moderate, predominantly localized in blood vessels and adjacent areas, reflecting the normal vascular supply of bone tissue. However, some stress-exposed groups, particularly G6, exhibited increased VEGF staining intensity, suggesting an enhanced angiogenic response possibly associated with bone remodeling under stress conditions. Lower magnification was used for VEGF images to identify the largest number of vessels in a field (Figure 8).

**Figure 8:**
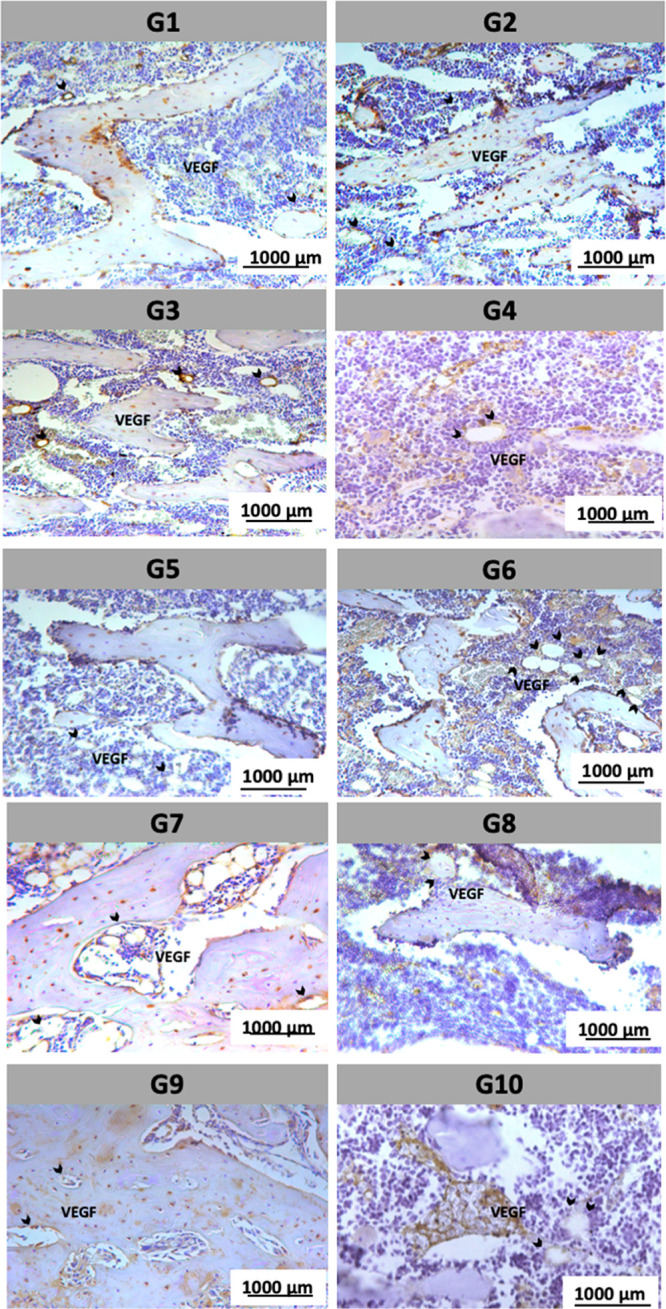
Immunohistochemical expression of VEGF. Moderate VEGF immunostaining was observed in the control groups (G1 and G2), predominantly localized in blood vessels and adjacent areas, reflecting the normal vascular supply of bone tissue. In contrast, some groups exposed to stress, particularly G6, showed increased staining intensity for VEGF, indicating an enhanced angiogenic response potentially associated with stress-induced bone remodeling. Black arrows indicate areas of positive VEGF staining. Scale bar: 1000 μm.

Furthermore, tartrate-resistant acid phosphatase (TRAP) staining was used to identify osteoclastic activity. A pinkish-red reaction, indicative of TRAP-positive osteoclasts, was observed more intensely in the groups subjected to the stress protocols. Positive cells were located primarily along the bone surface and in resorption lacunae, consistent with areas of active bone resorption. Specifically, TRAP activity was detected more intensely in the acute stress groups G4 (acute stress/normal rat) and G6 (acute stress/SHR), as well as in the chronic stress groups G8 (chronic stress/normal rat) and G10 (chronic stress/SHR), confirming an increase in osteoclastic activity under acute and chronic stress conditions (Figure 9).

**Figure 9:**
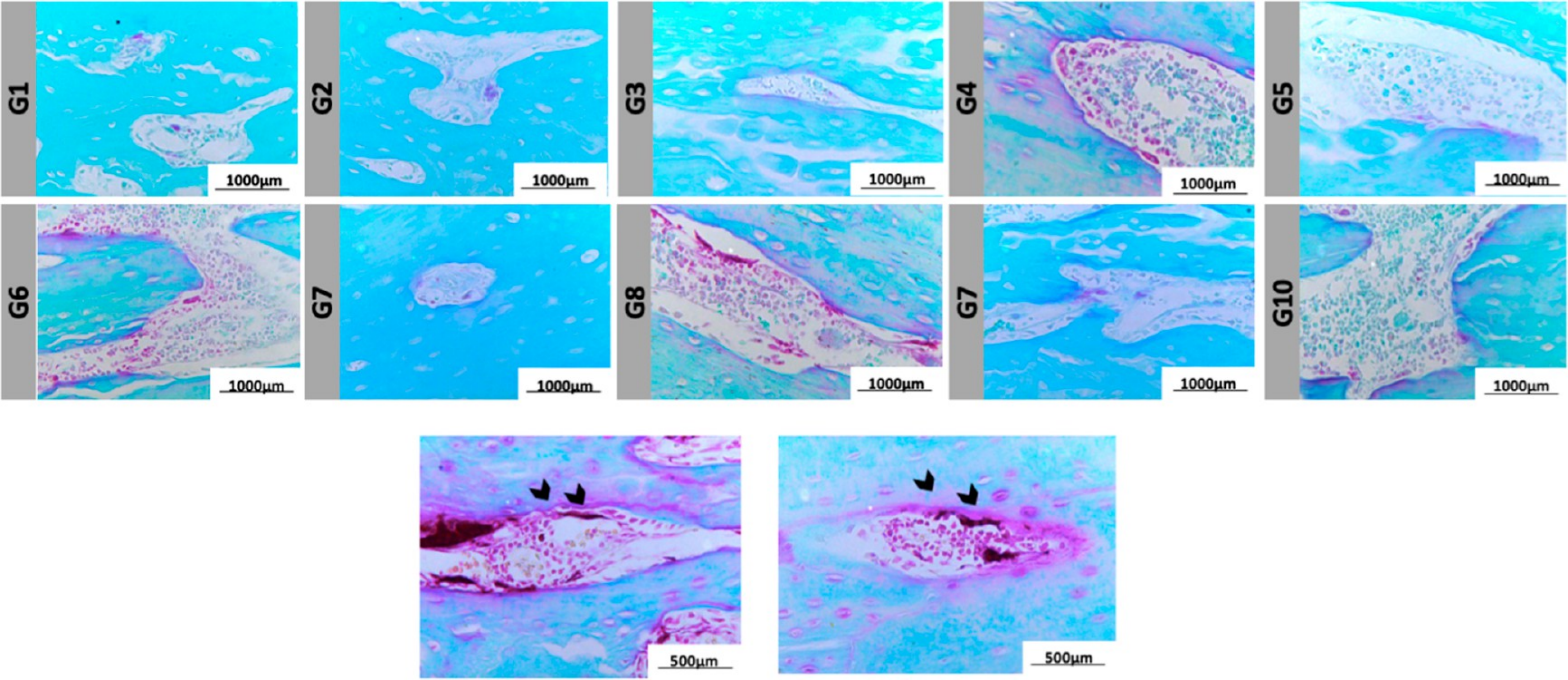
Tartrate-resistant acid phosphatase (TRAP) staining of osteoclasts from stress-exposed groups. A more intense pinkish-red reaction was detected in G4, G6, G8, and G10, with positive multinucleated cells located along the proximal bone surface. Black arrows highlight TRAP-positive osteoclasts in areas of resorption. Scale bar: 1000 μm.

These qualitative results indicate that stress influences the expression of key markers related to bone formation, vascularization and resorption.

### 3.4 Zymography Results (MMP-2 and MMP-9)

Gelatin zymography analysis was performed to evaluate the activity of matrix metalloproteinases MMP-2 (in pro, intermediate, and active forms) and MMP-9 in bone tissue from the different experimental groups (G1 to G10). Densitometric data of the corresponding bands were expressed in arbitrary intensity units.

The active form of MMP-2 showed the highest expression in groups G8 (0.875 ± 0.083) and G10 (0.775 ± 0.180), followed by G9 (0.575 ± 0.217), indicating increased gelatinolytic activity in these groups. In contrast, the lowest levels were observed in groups G2 (0.475 ± 0.171) and G3 (0.375 ± 0.050), suggesting reduced enzymatic activity.

The intermediate form of MMP-2 showed high expression in groups G1 (0.375 ± 0.129), G9 (0.375 ± 0.129), and G10 (0.400 ± 0.070), with the lowest expression in G4 (0.325 ± 0.103). These results suggest variations in intermediate enzymatic processing, possibly associated with the experimental treatment or the induced pathological condition.

Pro-MMP-2, the inactive form of the enzyme, showed similar levels across groups, with slight increases in G9 (0.400 ± 0.129) and G1 (0.375 ± 0.129), indicating a possible accumulation of the latent form without efficient conversion into the active form in some groups (Figure 10).

**Figure 10:**
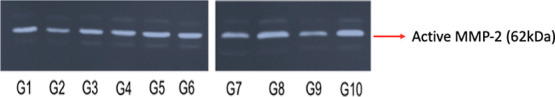
Gelatin zymography gel showing the expression of matrix metalloproteinase 2 (MMP-2) isoforms in experimental groups (G1–G10). An increased intensity of the active MMP-2 band is observed in groups G8 and G10, indicating higher proteolytic activity.

MMP-9 expression was generally lower than that of MMP-2 in all groups. However, a slight increase was observed in group G8 (0.225 ± 0.221), which presented the highest mean value, followed by G3 (0.225 ± 0.129) and G1 (0.175 ± 0.108). Group G4 showed the lowest expression levels (0.175 ± 0.095), suggesting possible inhibition of enzymatic activity in this group (Figure 11). Only the intermediate form is shown due to its detectable activity in bone tissue; active and pro-forms were negligible (Table 3) (Figure 12).

**Figure 11:**
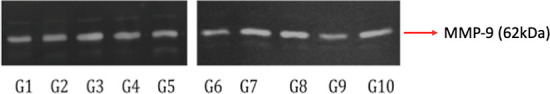
Representative zymography bands of MMP-9 expression in bone tissue from experimental groups (G1–G10). Overall, MMP-9 expression was lower than that of MMP-2 in all groups. A slight increase was observed in group G8 (0.225 ± 0.221), which presented the highest mean value, followed by G3 (0.225 ± 0.129) and G1 (0.175 ± 0.108). Group G4 exhibited the lowest expression levels (0.175 ± 0.095), suggesting possible inhibition of enzymatic activity in this group.

**Table 3:** Densitometric Analysis of MMP-2 and MMP-9 Expression (Arbitrary Units of Intensity)^a^

group	active MMP-2 (62 kDa)	intermediate MMP-2 (64 kDa)	Pro-MMP-2 (68 kDa)	MMP-9
G1	0.525 ± 0.158	0.375 ± 0.129	0.375 ± 0.129	0.175 ± 0.108
G2	0.475 ± 0.171	0.350 ± 0.152	0.350 ± 0.152	0.150 ± 0.050
G3	0.375 ± 0.050	0.350 ± 0.100	0.350 ± 0.100	0.225 ± 0.129
G4	0.525 ± 0.175	0.325 ± 0.103	0.325 ± 0.103	0.175 ± 0.095
G5	0.525 ± 0.100	0.350 ± 0.050	0.350 ± 0.050	0.150 ± 0.050
G6	0.500 ± 0.200	0.350 ± 0.150	0.350 ± 0.150	0.150 ± 0.100
G7	0.550 ± 0.129	0.350 ± 0.050	0.350 ± 0.050	0.150 ± 0.050
G8	0.875 ± 0.083	0.350 ± 0.050	0.350 ± 0.050	0.225 ± 0.221
G9	0.575 ± 0.217	0.375 ± 0.129	0.400 ± 0.129	0.150 ± 0.100
G10	0.775 ± 0.180	0.400 ± 0.070	0.375 ± 0.129	0.200 ± 0.100

a Results are expressed as mean ± standard deviation in arbitrary intensity units.

**Figure 12:**
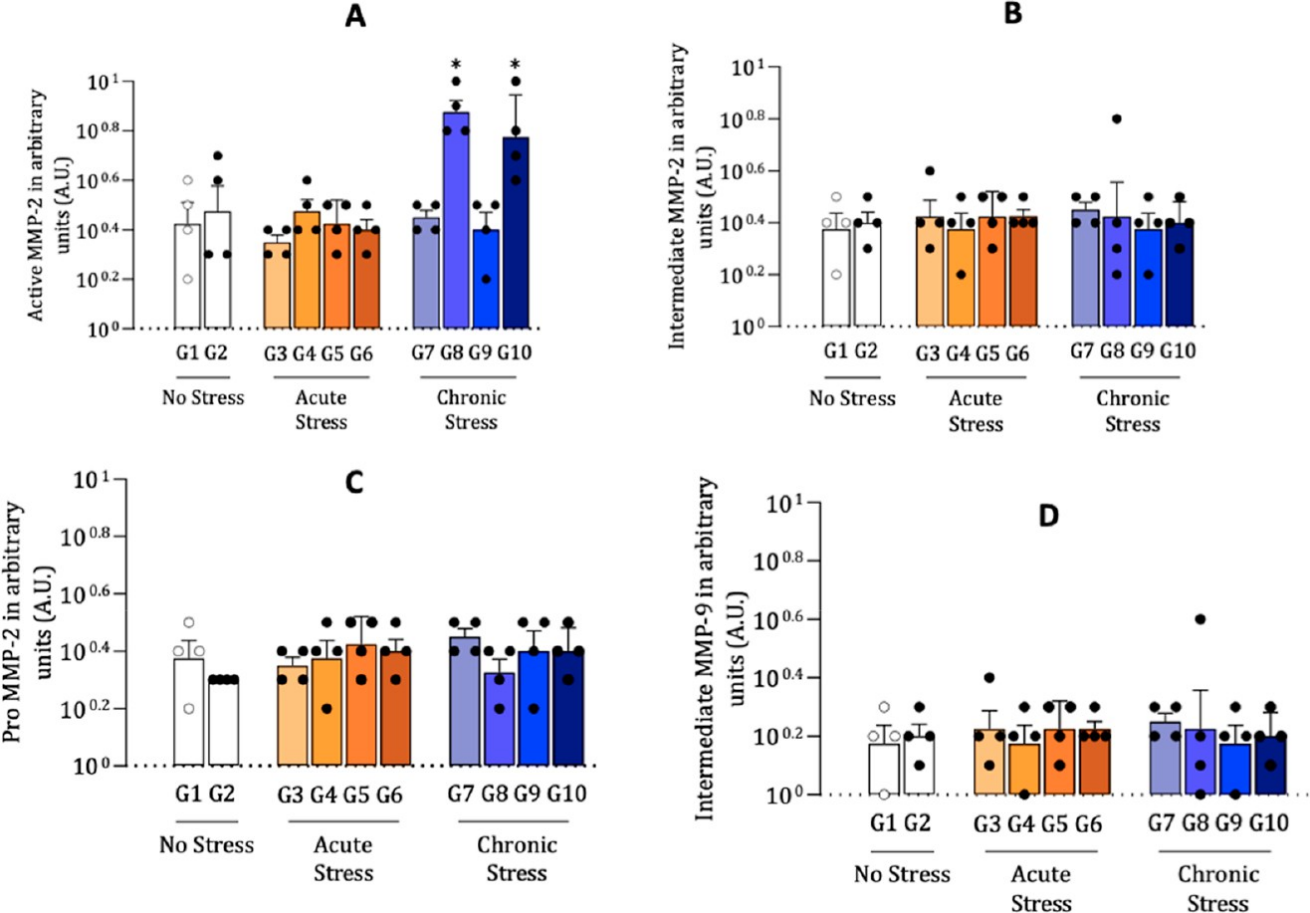
Densitometric quantification of gelatin zymography for matrix metalloproteinases (MMPs) in experimental groups. Values are presented as mean ± standard deviation. (A) Active MMP-2: significantly increased in G8 and G10 compared to their respective controls. (B) Intermediate MMP-2: expression was generally homogeneous; however, G8 showed a significant reduction compared to G7. (C) Pro-MMP-2: slight increases were observed in G9 and G10. Specifically, G9 was elevated compared to G2 but not to G5, G7 was increased relative to G1 and G3, and G10 was comparable to G5. (D) MMP-9 (intermediate form): overall low expression across groups, with minor elevations in G3 and G8. For MMP-9, only the intermediate form was detectable in bone tissue, whereas active and pro-forms were negligible. Analyses were performed in duplicate (*n* = 6 replicates per group). Asterisks indicate statistical significance (*p* < 0.05).

## 4 Discussion

In this study, the impact of acute and chronic stressors on bone characteristics in spontaneously hypertensive rats (SHR) and normotensive controls using quantitative histology, immunohistochemistry, and zymographic analyses. Despite significant physiological stress responses indicated by elevated corticosterone levels in both acute and chronic stress groups, quantitative bone histomorphometry and type I collagen fiber density remained largely unchanged across groups. These findings suggest that, within the experimental time frame, stress exposure did not significantly alter overall bone mass or collagen content, consistent with previous studies reporting that short-term or moderate stress may not directly affect bone volume or matrix composition.^35,36^

The highest corticosterone concentrations were observed in SHR subjected to acute stress (G6:71.3 ± 15.3 ng/mL), reflecting strong activation of the hypothalamic–pituitary–adrenal (HPA) axis. This aligns with previous evidence showing that SHRs exhibit enhanced ACTH (adrenocorticotropic hormone) responses to acute stressors such as cold or immobilization compared to normotensive rats, although corticosterone levels may vary depending on stressor type and methodological conditions.^37^ More broadly, HPA axis activation and glucocorticoid release represent hallmark adaptive responses to acute physical or emotional stress.^38^ Thus, the pattern observed in G6 likely reflects an adaptive glucocorticoid response, although its magnitude appears strain- and stressor-dependent. From a chemical perspective, the elevated corticosterone concentrations observed under acute stress highlight the dynamic regulation of steroid hormone biosynthesis in SHR. Corticosterone is synthesized from cholesterol via tightly regulated enzymatic steps involving cytochrome P450 enzymes in the adrenal cortex, and acute HPA axis activation accelerates these reactions, increasing flux through steroidogenic pathways and glucocorticoid accumulation in circulation. This enhanced steroidogenic activity increases the demand for reducing equivalents (e.g., NADPH) and may affect redox balance and mitochondrial function in adrenal cells. Additionally, corticosterone modulates systemic glucose, lipid, and protein metabolism through glucocorticoid receptor–mediated transcriptional mechanisms, linking acute stress exposure to downstream metabolic regulation.^39^

Quantitative histomorphometric analysis of trabecular bone (BA/TA, %) revealed mean values between 8.54 and 10.85%, without statistically significant differences among groups. This suggests that neither acute nor chronic stress, within the studied time frame, was sufficient to induce measurable changes in trabecular bone mass. However, a slight difference was observed between groups G2 and G9, which may be attributed to the variability within groups, as reflected by the standard deviation. Literature supports that glucocorticoid-induced or stress-related bone loss often becomes apparent only after prolonged exposure and is more sensitively detected using three-dimensional micro-CT or dynamic histomorphometry.^40,41^ In fact, in our previously published study using micro-CT,^29^ significant alterations in trabecular parameters were detected between specific groups (e.g., G1 vs G8; G4 vs G5), despite the absence of differences in conventional histological analysis. These findings emphasize the higher sensitivity of micro-CT to capture early and subtle microarchitectural alterations that two-dimensional histomorphometry may overlook.

Although a statistically significant difference was detected between G2 and G9. Thus, some visual overlap does not preclude the presence of statistical significance, as confirmed by the ANOVA followed by Tukey’s post hoc test. However, the magnitude of this difference was small and, from a biological perspective, does not reflect a consistent or relevant effect on bone formation. This finding highlights the need to interpret statistical outcomes in conjunction with biological plausibility.

Thus, while histology provides essential qualitative and quantitative information on bone tissue, its limited resolution in detecting microstructural changes reinforces the need for complementary methods. Indeed, chronic glucocorticoid treatment has been shown to suppress bone formation and impair mechanical strength even in the absence of short-term reductions in trabecular volume.^42^ Taken together, these results indicate that the absence of significant histological differences should be interpreted with caution, as bone alterations may be underestimated when relying exclusively on traditional histomorphometric approaches.

Total collagen fiber quantification, assessed by Picrosirius Red staining under polarized light, showed mean values between 7.15% (G4) and 9.72% (G3). The normal control group (G1) recorded 9.71%, and the SHR control group (G2) 9.33%, with overall comparable values across groups. However, a statistically significant difference was observed between G7 (chronic stress/control) and G8 (chronic stress), suggesting that chronic stress exposure subtly affected collagen fiber content in normal rats. These findings suggest that the transient increase in corticosterone was insufficient to promote detectable remodeling of the collagen matrix at this stage. Similar observations have been reported in periodontal models, where disorganization and thinning of collagen fibers became evident only after prolonged periods of functional hypostimulation.^43–46^ Nonetheless, it is important to acknowledge the methodological limitations of Picro Sirius Red, which provides an overall estimate of collagen density but does not allow precise evaluation of fibrillar maturation or the detection of early molecular changes. Such subtle alterations would require complementary approaches, including immunohistochemistry or biochemical assays, to fully elucidate stress-induced effects on collagen turnover.^47^

Although numerical variations in collagen fiber content were observed among the groups, statistical analysis confirmed that only the comparison between groups G7 and G8 reached significance. The differences noted between G3 and G4, as well as between G9 and G10, did not achieve statistical significance, indicating that these variations likely reflect biological variability rather than consistent effects of the experimental interventions. This reinforces that the protocols tested did not broadly impact collagen density, with the exception of the discrete alteration observed in G7 versus G8.

Immunohistochemical analyses, however, revealed subtle molecular effects of stress. Osteocalcin (OCN), a late osteoblast differentiation marker, showed reduced expression in stress-exposed groups, suggesting impaired osteoblast activity and mineralization potential. Similarly, bone sialoprotein (BSP), a critical nucleator of hydroxyapatite, also presented diminished expression, reflecting compromised matrix quality under stress. These results align with previous studies showing that stress and glucocorticoids suppress osteoblast differentiation and reduce noncollagenous protein expression, contributing to impaired bone formation.^48–52^

The reduced osteocalcin expression observed in stress-exposed groups suggests that stress interferes with bone formation primarily by impairing osteoblast functional activity rather than simply reducing cell number. As osteocalcin plays a key role in matrix maturation and mineral deposition, its downregulation indicates a diminished capacity for proper mineralization, which may result in structurally weaker bone tissue. In this context, stress-induced glucocorticoid signaling likely disrupts osteoblast differentiation and function, leading to the production of a matrix with inferior mineral quality. Moreover, the presence of osteocalcin in osteocytes implies that stress-related reductions in its expression may also affect osteocyte-mediated regulation of bone remodeling, further exacerbating the negative impact of stress on bone homeostasis.^58^

In contrast, vascular endothelial growth factor (VEGF) expression was increased in certain stress groups, particularly G6, indicating a possible compensatory angiogenic response. This adaptive upregulation of VEGF may aim to sustain bone perfusion and remodeling under adverse conditions.^48–59^ Together, these findings suggest that stress suppresses osteoblastic activity (OCN, BSP) while promoting angiogenesis (VEGF), potentially creating an imbalance between bone formation and vascular support.^60^

Zymography analysis revealed important differences in matrix metalloproteinase activity, particularly MMP-2, among the experimental groups. The active form of MMP-2 was significantly increased in groups G8 and G10, suggesting intensified proteolytic activity in these animals. Since MMP-2 is involved in the degradation of type IV collagen and matrix remodeling, this finding may indicate enhanced bone remodeling in these groups, possibly related to the effect of the experimental treatment. Conversely, the lower levels of active MMP-2 observed in G2 and G3 may reflect reduced enzymatic activity, consistent with lower remodeling or a less dynamic bone environment. The intermediate form of MMP-2 showed relatively homogeneous expression across groups, with slight variations, suggesting that partial processing of the enzyme occurs consistently in bone tissue, regardless of treatment. The slight increase observed in G10 may represent greater availability of the enzyme for conversion into its active form. Similarly, the discrete elevation of pro-MMP-2 in some groups, especially G9, may indicate accumulation of the latent form, possibly due to reduced efficiency of its activation. Regarding MMP-9, its expression was generally lower compared to MMP-2, which is consistent with previous reports describing MMP-9 as less prominent in bone under physiological conditions. The slight increase in G8 and G3 may reflect an adaptive response to higher remodeling demand, as MMP-9 plays a critical role in osteoclast-mediated bone resorption. Overall, these findings suggest that the treatment influenced bone remodeling dynamics by modulating metalloproteinase activity. The increase in the active form of MMP-2 in certain groups may be associated with greater remodeling and reorganization of the matrix, while the changes in MMP-9, although modest, reinforce its complementary role in bone resorption. These results support the hypothesis that the balance between the active, intermediate, and pro-forms of MMPs is crucial for maintaining bone integrity and can be modulated by metabolic factors.^61–68^

This suggests that stress can induce early alterations in bone remodeling even in the absence of detectable changes in bone mass. Previous studies corroborate these results, showing that stress may increase osteoclast activity and affect bone homeostasis through modulation of molecular pathways controlling osteoclast differentiation and function.^69,70^ Taken together, the present results demonstrate that acute and subchronic stressors, although insufficient to reduce bone volume or collagen density, negatively modulate osteoblast function and matrix protein expression while enhancing enzymatic activity and angiogenic signaling. These molecular changes may precede structural bone alterations and, if sustained, could compromise long-term bone quality, particularly relevant in hypertensive individuals, who already present a predisposition to bone fragility. Some limitations should be acknowledged. The use of SHRs, a genetically hypertensive strain, may limit extrapolation to normotensive models. The relatively short experimental period might have prevented detection of cumulative effects, and analysis restricted to tibial bone does not account for site-specific skeletal variations.

Moreover, while immunohistochemistry provided qualitative insights, future studies should incorporate quantitative analyses of osteoblast and osteoclast numbers, trabecular microarchitecture, and biochemical assays. Finally, physiological stress markers beyond corticosterone (e.g., heart rate variability, behavioral parameters) could enhance validation of systemic responses. From a clinical perspective, these findings reinforce the relevance of stress management strategies in patients with hypertension, who already present a higher risk of bone fragility. Early molecular alterations such as reduced osteoblast activity, enhanced osteoclast resorption, and increased MMP activity may precede detectable reductions in bone density, suggesting that conventional bone assessments might underestimate the impact of psychosocial stress.

Therefore, interventions aimed at controlling psychological stress, such as behavioral therapies, physical exercise, or pharmacological approaches, could represent an additional preventive strategy to preserve bone health in hypertensive populations. Despite these constraints, this study provides relevant evidence that psychosocial stress influences bone metabolism at the molecular level, modulating osteoblast function, angiogenesis, and matrix remodeling enzymes. These findings underscore the importance of integrative approaches combining molecular, histological, and physiological analyses to fully understand the skeletal consequences of stress exposure.

## 5 Conclusions

In conclusion, acute and subchronic stressors did not significantly affect trabecular bone volume or collagen density within the studied time frame, but they negatively modulated osteoblast function (OCN, BSP), increased enzymatic remodeling activity (MMP-2, MMP-9), and promoted angiogenic signaling (VEGF). Importantly, TRAP-positive osteoclasts were detected exclusively in stress (exposed groups, highlighting a stress) specific increase in bone resorption, particularly in the hypertensive (SHR) model, which represents a high-risk population. Thus, the results of corticosterone dosage, as well as histological and immunohistochemical analyses, reinforce that stress promotes changes in bone metabolism, which promote structural alterations and reveal a possible mechanism by which stress can compromise bone quality.

## Supplementary Material



## Notes

Annex A contains the individual characterization of all experimental animals included in the study (R1–R40). The table details group allocation (G1–G10), experimental condition (baseline, acute, and chronic protocols; control and stress), strain (normotensive or SHR), body weight (g), and arterial pressure (mmHg) values for each animal (PDF)
