# Supplementary material for: Psychological Stress Modulates Bone Remodeling Pathways in Normotensive and Hypertensive Rats: A Cellular and Molecular Approach
Source: ACS Omega. 2026 Feb 19;11(8):13558–71. doi: 10.1021/acsomega.5c11197 (PMC12961481; doi:10.1021/acsomega.5c11197)
Supplement: Supplementary file 1 [file ao5c11197_si_001.pdf]

Psychological Stress Modulates Bone Remodeling Pathways in Normotensive and Hypertensive Rats: A Cellular and Molecular Approach

Marina Ribeiro Paulini [1], Dimitrius Leonardo Pitol [1], Glauce Crivelaro do Nascimento [1], Daniela Vieira Buchaim [2,3,4], Marcelo Rodrigues da Cunha [5], Rogerio Leone Buchaim [7] and João Paulo Mardegan Issa [1,]*

[1] Department of Basic and Oral Biology, School of Dentistry of Ribeirão Preto, University of São Paulo (FORP-USP), Ribeirão Preto 14040-904, Brazil; marina.paulini@usp.br; dimitrius@forp.usp.br; glauce.nascimento@usp.br

[2] Medical School, University Center of Adamantina (FAI), Adamantina 17800-000, Brazil; danibuchaim@alumni.usp.br

[3] Graduate Program in Anatomy of Domestic and Wild Animals, Faculty of Veterinary Medicine and Animal Science, University of São Paulo (FMVZ-USP), São Paulo 05508-270, Brazil

[4] Department of Postgraduate, Dentistry School, Faculty of the Midwest Paulista (FACOP), Piratininga 17499-010, Brazil

[5] Postgraduate Program in Health Sciences, Faculty of Medicine of Jundiaí (FMJ), Jundiaí 13202-550, Brazil; marcelocunha@g.fmj.br

[7] Department of Biological Sciences, Bauru School of Dentistry (FOB/USP),

University of Sao Paulo, Bauru 17012-901, Brazil; rogerio@fob.usp.br

Annex A- Experimental animals (R1–R40)

Animal	Group	Condition	Strain	Body weight (g)	Arterial pressure (mmHg)
**R1**	G1	Baseline control	Normotensive	248.9	11.2 × 7.5
**R2**	G1	Baseline control	Normotensive	250.4	11.5 × 7.7
**R3**	G1	Baseline control	Normotensive	251.1	11.6 × 7.8
**R4**	G1	Baseline control	Normotensive	249.6	11.3 × 7.6
**R5**	G2	Baseline control	SHR	248.7	17.2 × 11.1
**R6**	G2	Baseline control	SHR	250.1	17.5 × 11.4
**R7**	G2	Baseline control	SHR	249.3	17.6 × 11.5
**R8**	G2	Baseline control	SHR	250.6	17.8 × 11.6
**R9**	G3	Acute control	Normotensive	249.8	11.4 × 7.6
**R10**	G3	Acute control	Normotensive	250.9	11.6 × 7.8
**R11**	G3	Acute control	Normotensive	251.4	11.7 × 7.9
**R12**	G3	Acute control	Normotensive	250.1	11.5 × 7.7
**R13**	G4	Acute stress	Normotensive	250.2	12.9 × 8.3
**R14**	G4	Acute stress	Normotensive	251.7	13.2 × 8.5
**R15**	G4	Acute stress	Normotensive	252.1	13.4 × 8.6
**R16**	G4	Acute stress	Normotensive	250.9	13.0 × 8.4
**R17**	G5	Acute control	SHR	249.5	17.4 × 11.2
**R18**	G5	Acute control	SHR	250.8	17.7 × 11.5
**R19**	G5	Acute control	SHR	251.2	17.8 × 11.6
**R20**	G5	Acute control	SHR	249.9	17.5 × 11.3
**R21**	G6	Acute stress	SHR	250.6	18.6 × 12.1
**R22**	G6	Acute stress	SHR	251.9	18.9 × 12.3
**R23**	G6	Acute stress	SHR	252.4	19.2 × 12.5
**R24**	G6	Acute stress	SHR	251.0	18.7 × 12.2
**R25**	G7	Chronic control	Normotensive	250.7	11.5 × 7.7
**R26**	G7	Chronic control	Normotensive	251.9	11.7 × 7.9
**R27**	G7	Chronic control	Normotensive	252.3	11.8 × 8.0
**R28**	G7	Chronic control	Normotensive	251.2	11.6 × 7.8
**R29**	G8	Chronic stress	Normotensive	244.3	13.3 × 8.7
**R30**	G8	Chronic stress	Normotensive	245.9	13.6 × 8.9
**R31**	G8	Chronic stress	Normotensive	246.4	13.8 × 9.1
**R32**	G8	Chronic stress	Normotensive	245.1	13.4 × 8.8
**R33**	G9	Chronic control	SHR	250.9	17.6 × 11.4
**R34**	G9	Chronic control	SHR	251.8	17.9 × 11.7
**R35**	G9	Chronic control	SHR	252.2	18.1 × 11.8
**R36**	G9	Chronic control	SHR	251.3	17.8 × 11.6
**R37**	G10	Chronic stress	SHR	244.7	19.3 × 12.9
**R38**	G10	Chronic stress	SHR	245.9	19.7 × 13.2

**R39**	G10	Chronic stress	SHR	246.6	20.0 × 13.4
**R40**	G10	Chronic stress	SHR	245.2	19.5 × 13.0
